# Survey data on key climate and environmental drivers of farmers’ migration in Burkina Faso, West Africa

**DOI:** 10.1016/j.dib.2016.11.001

**Published:** 2016-11-09

**Authors:** Safiétou Sanfo, M. William Fonta, Ibrahim Boubacar, P.A. John Lamers

**Affiliations:** a West African Science Service Center on Climate Change and Adapted Land Use (WASCAL), Bvd Mohamad Kadhafi 06, BP 9507 Ouagadougou 06, Burkina Faso; b Center for Development Research (ZEF), Walter Flex-Straße 3, 53113 Bonn, Germany

**Keywords:** Climate and environmental changes, Climate and environmental drivers, Environmentally induced migration, West Africa

## Abstract

This article describes two datasets generated from various sources in south western Burkina Faso to identify the key climate and environmental drivers that cause farmers to migrate. The survey sampling is random but reasoned and rational. The first dataset from 367 farm households^1^ contains data on farmers’ perception of climate change risks or hazards, their impacts on farmland productivity and farm households’ risk management strategies. The second dataset from 58 farm households contains data on agricultural practices, environmental changes, and environmental migration. Three supplemental Excel sheets show the results of the surveys. Details on the sample as well as further interpretation and discussion of the surveys are available in the associated research article (‘Field Facts for Crop Insurance Design: Empirical Evidence from South Western Burkina Faso’ (W. M. Fonta, S. Sanfo, B. Ibrahim, B. Barry, 2015) [1]).

**Specifications Table**

**Table:** 

Subject area	Economics
More specific subject area	Environmentally-induced migration
Type of data	Excel file, figures
How data was acquired	Survey, guided interviews and focus group discussions
Data format	Raw, analyzed
Experimental factors	The selected farm household have more than twenty years’ farming experience and are indigenous or migrant farmers.
Experimental features	Farm households’ perception of climate and environmental changes, soil fertility decline, soil productivity decline, and land degradation. Soil fertility and productivity are considered to be pull factors in the host zone. Deforestation and land degradation are major push factors in the source region.
Data source location	Ouagadougou, Burkina Faso.
Data accessibility	Data is with this article

**Value of the Data**

• Researchers can examine the raw data on farm households’ perception of climate hazards and key environmental factors that cause farmers to migrate using statistical methods such as descriptive statistics, analysis of variance, linear regression, etc. However, any statistical analysis must be done with caution as the sample is relatively small.
• Findings from the dataset are included. Analyses of these data allow for comparisons between this sample and parallel samples in other similar studies elsewhere, namely in other West African countries.
• The key variables in farm households’ perception of climate risk and environmental changes as well as key environmental variables that cause farmer to migrate were captured through semi-structured interviews, life histories and focus group discussions. Analyses of these data generated through such a conceptual framework may be compared to analyses of other dataset collected using alternative conceptual frameworks.

## 1 Data

**Fig. 1:**
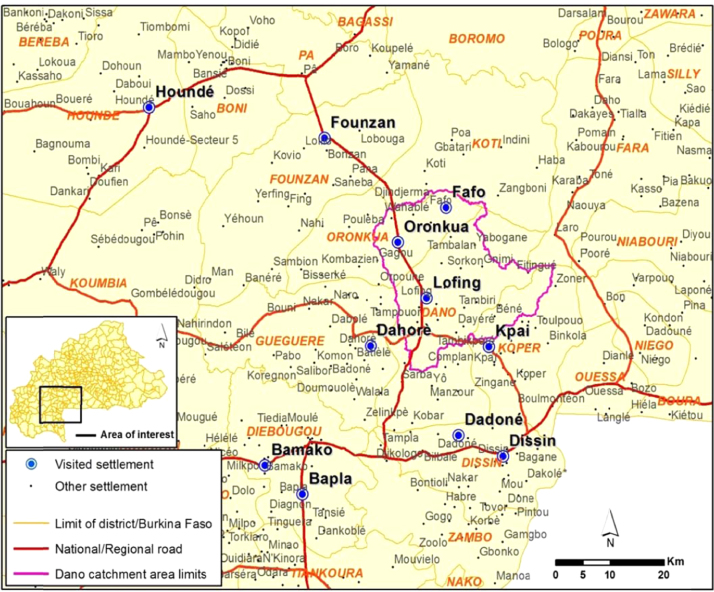
Visited settlements.

Data were collected from the southern region of Burkina Faso (Fig. 1 and Table 1). This region is located between the 700 and 1100 mm isohyets and subject to severe dry spells in the rainy season [2]. Agriculture and livestock are the main subsistence activities for the rural population, which makes up about 88% of the total population of 620,767 individuals [3]. The southern region is seen as blessed with favorable rainfall conditions, and the Burkina Faso agricultural services classify this region as the country׳s breadbasket. However, several studies revealed that, while the area is suitable for crop production, it has been affected by climate change. Hence, this southern region was selected as it represents an environment that has contrasting agricultural potential but still has some of the highest agricultural potential in the country.

The dataset from the 100 farm household survey (93 men and 7 women heads of household), collected through a questionnaire survey from May to June 2014, describes variables such as family size, gender of the respondent, the head of household׳s level of education, knowledge about climate hazards, crop damage caused by recent climate hazards, etc. The average age of surveyed farmers is about 48 years old.

The focus group discussions with 267 farmers (241 men and 26 women heads of household), performed from May to June 2014, include local indicators of climate change and its variability, climate risks to agriculture, agricultural finance, and discussions on local strategies to adapt to climate change and food scarcity. The dataset from the 58 farm household semi-structured interviews (57 men and 1 woman heads of household), collected from March 23 to 29, 2015, investigated agricultural practices, farmers’ perception of environment risks, and key environmental drivers that cause farm households to migrate. Two Excel sheets summarize the variables contained in the datasets.

## 2 Experimental design, materials and methods

### 2.1 Experimental design

The first dataset (Excel sheet 1) contains the findings from the 100 farm household survey. This represented a selected sample of the Burkina Faso Ministry of agriculture as well as Planet Guarantee (an insurance company) pilot project on crop insurance. We designed a set of questionnaires to perform the survey [1]. We divided the items in the questionnaires into four sections. Section **I** provided demographic data including the respondents’ age, gender, education and marital status, and sub-items pertaining to farm household size, ethnicity and religion. Section **II** described farm household employment including each farm household׳s principal function, farm activities and off-farm activities. Section **III** evaluated farmers’ perception of climate hazards. It included items such as the number of years that the farm households had been farming, whether the farm households had heard of climate risk, and how the farmer could explain climate hazards as well as how they compared climate risks to other risks (with 1=the worst, 2=worse, 3=very moderate, 4=moderate, and 5=do not know). Section **IV** dealt with the effects of climate change. It included questions about season onset and offset, dry spell frequency, dry spell damage to crops in relation to growing stage (sowing, flowering and harvesting), and the principal factors that cause “bad” crop yields. We generated the focus group discussion data from the 267 farmers using an interview guide. These FGDs were conducted to gather farmers with similar backgrounds/experiences in order to discuss a specific topic of interest. In bridging research and policy, the FGD can be useful in providing an insight into different opinions among different parties involved in the change process. The group discussions revolved around three main points, namely: local indicators of climate change, climate risks to agriculture, and local strategies to adapt to climate change and food scarcity.

**Fig. 2:**
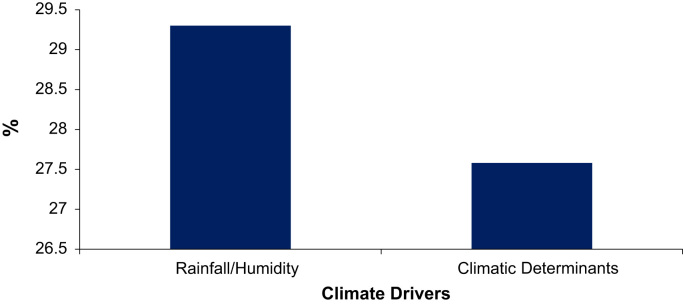
Climate factors affecting farmers’ migration decisions in Southern Burkina Faso.

**Fig. 3:**
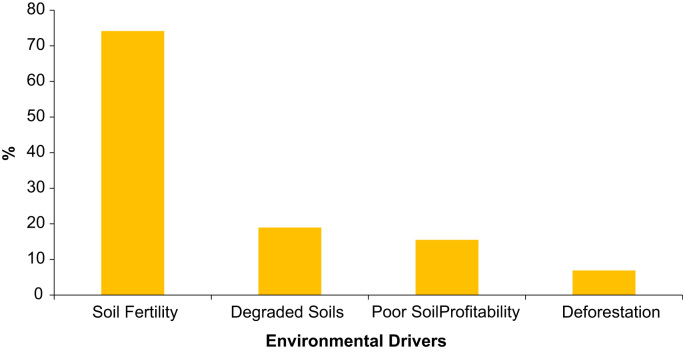
Environmental factors affecting farmers’ migration decisions in Southern Burkina Faso.

**Fig. 4:**
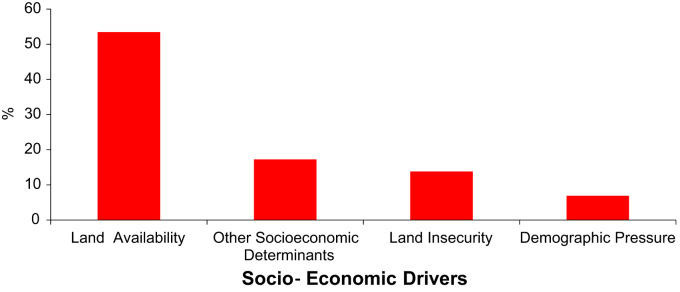
Socio-economic factors affecting farmers’ migration decisions in Southern Burkina Faso.

Excel sheets 2 and 3, Fig. 2, Fig. 3, Fig. 4, contain a dataset generated through the 100 farm household survey and a semi-structured survey of 58 farm households. We surveyed these 58 households as a supplement to the 100 households. Items include agricultural and land use practices, soil fertility and the need for adapted land use, and climate and environmental conditions in the source and host zones of migrant farmers. Farmers were also asked questions on the difference between native and migrant farmers, the factors that motivate farmers’ migration and the migration process (group/individual migration), as well as on the development of the agricultural sector over the short, medium and long term.

### 2.2 Materials

To collect the survey data, we designed a written questionnaire. We held focus group discussions (FGDs) to collect detailed qualitative information (life histories and focus group discussion data) using a discussion guide. Finally, we used guided interviews to collect semi-structured data.

### 2.3 Method

Two research assistants and eight graduate students from the University of Ouagadougou were used to perform the survey. They were selected based mostly on statistics competency and local language fluency. The interviewers received three days of intensive training covering each aspect of the questionnaire as well as training on the household mapping process using a global positioning system (GPS). To perform the survey efficiently, we divided the team of interviewers into groups of two to resolve the language barrier issue and the lack of GPS equipment (only 5 GPS units for ten people). In addition, we wanted to prevent other potential forms of bias such as enumerator bias and sampling non-response bias. We also agreed that all team members would take part in the administration of the first survey questionnaire to facilitate their understanding of it and identify potential difficulties that might arise during the course of the study.

To facilitate the field work in Southern Burkina, the *Direction Provincial de l’ Agriculture* (Provincial Agriculture Department) was used to initiate pre-survey contacts with the selected villages in the region. A total of 10 villages were selected for the farm household survey. The selected villages needed to be close to operating meteorological stations in the region to make it possible to validate farmers’ reports with monitored standard meteorological information. This was part of the first study we conducted on farmers’ perceptions and meteorological data with the aim of comparing farmers’ perceptions with historical meteorological data.

In each of the selected villages, 10 households were purposefully selected. The main selection criterion was enough years of farming experience to at least have sufficient knowledge of climate change and its variability. The boundary condition was that the farmer had more than twenty years’ farming experience. In each of the selected villages, we met with the chair of the *Comité Villageois pour le Development* (CVD, the Local Development Committee) to explain the purpose of the survey and obtain a formal description of the village and its organizational structure. As indicated earlier, all team members participated in the interview of the head of the first household selected in the first village. The first interview lasted for over 4 hours. After this test survey, the team surveyed the rest of the selected households in each selected village. This was followed by FGDs in each of the selected villages.

To perform the semi-structured survey as a supplement to the 100 households, we first contacted the head of the *Direction Provincial de l’Agriculture* (Provincial Agriculture Department) who helped choose two relevant villages. The selected villages are located in the least populated zones of the region and are generally recognized as having few native inhabitants (the Pougoulis). Due to the availability of cropland and lowland assets (fertility and humidity), this selected zone has long been a draw zone for migrants, especially farmers. In both villages, indigenous and migrant farmers were selected as research interviewees. We spoke with the chair of the Local Development Committee to select the farmers to be surveyed. The selected farmers were both native to the area and migrants. A total of 58 households were surveyed.

We used the Statistical Package for the Social Sciences (SPSS), MAXQDA (a software program designed for computer-assisted qualitative and mixed method data and text analysis), and MS Excel to process and analyze the data. While MAXQDA only processes entered data, SPSS and MS Excel were used for statistical purposes. The statistics are mainly descriptive, including mean values, standard deviations, and frequency distributions where applicable. The decision to use the qualitative with quantitative methods in conjunction with each other was driven by the goal of understanding specific aspects. For instance, qualitative methods were used to explore the understanding of the quantitative survey findings that could not be cross-checked using statistics.

## Figures and Tables

**Table 1:** Demographic climate and environmental information on the study area.

Variables		Visited Villages										
		Bamako	Bapla	Dadoné	Dahoré	Fafo	Founzan	Houndé	kpai	Lofing	Oronkua	**Total**
Population		**1 325**^a^	**1 677**^a^	**917**^a^	**830**^a^	**3 500**^a^	**45 750**^a^	**21 830**^a^	**2 450**^a^	**1 423**^a^	**26 952**^a^	
Respondents	Quantitative survey (from 28 May to 5 June 2014)	10	10	10	10	10	10	10	10	10	10	**100**
	Focus group discussion (from 28 May to 5 June 2014)	23	22	16	51	29	47	19	15	22	23	**267**
	Qualitative survey (from March 23 to 29, 2015)	6	5	6	6	6	6	6	6	6	5	**58**
		
Climatic and environmental variables (South Western region)	Rainfall (mm)	903^b^										
	Temperature (Min; Max) (^o^C)	21.2 ; 33.4^b^										
	Sunshine (hours)	7.7^b^										
	Wind Speed (m/s)	1.8^b^										
	Humidity (Min; Max) (%)	34.5; 35.2^b^										
	Vegetation	Common tree species are shea trees (*Vitellaria paradoxa*, Sapotaceae), *Parkia biglobosa* (Mimosacées), *Balanites aegyptiaca* (Balanitaceae), and *Piliostigma thonningii* (Fabaceae - Caesalpinioideae). Woodland is generally observed in old fallow. In more recent fallow, a savannah occurs frequently. Along the rivers, the vegetation is a gallery forest. A mosaic of shrub is observed on hills where combretum occur frequently. The grass layer is dominated by tall, perennial grass species such as *Andropogon gayanus*, *Loudetiopsis kerstingii, Cymbopogon schoenanthus* , *Andropogon pseudapricus*, *Pennisetum pedicellatum*.										
	Soil Type	• Tropical eutrophic brown soils on clay material. The chemical potential is high. They are the best soils in the country; • Tropical leached ferruginous soils on sandy material, these are soils with low agronomic value, spatially dispersed; • Ferrallitic soils on sandy-clayey material: these are permeable, acidic soils with low chemical potential; • Mineral hydromorphic and pseudogley soils on varied texture material. These soils are favourable to many crops; their chemical potential is average; • Raw mineral soils associated with slightly developed soils. Their agronomic interest is low or zero. These are mainly land reserved for grazing; • Vertisoils and eutrophic brown soils. These are average to high agronomic value soils, suitable for all crops in the region. They can be easily improved; • Tropical ferruginous soils. They have a very low agronomic value and can only be used for undemanding subsistence crops such as fonio and millet; • Hydromorphic soils, located in lowlands and flooding areas. These are heavy, but with high agronomic value. They are excellent lands for gardening.										

a INSD Projection, 2016;

b Burkina Faso Meteorological Department (Average 1970–2013).

## References

[1] W.M. Fonta, S. Sanfo, B. Ibrahim, B. Barry, Field facts for crop insurance design: empirical Evidence from Southern Burkina Faso, in: Accelerating Research on Innovations for Rural Financial Inclusion in Africa. Working paper series – African Microfinance Week, 2015, pp. 58–65

[2] Ibrahim B., Polche J., Karambiri H., Rockel B. (2012). Characterization of the rainy season in Burkina Faso and its representation by regional climate models. Clim. Dyn..

[3] INSD Recensement Général de la Population et de l’Habitat (RGPH). Rapport définitif (2012), Ouagadougou, Burkina Faso.

