# Supplementary material for: Survey data on key climate and environmental drivers of farmers’ migration in Burkina Faso, West Africa
Source: Data Brief. 2016 Nov 9;9:1013–9. doi: 10.1016/j.dib.2016.11.001 (PMC5122697; doi:10.1016/j.dib.2016.11.001)
Supplement: Supplementary file 1 — Supplementary material [file mmc1.docx]

**Conflict Of Interest Form.**

Conflicts of interest: none
