# Supplementary material for: Survey data on key climate and environmental drivers of farmers’ migration in Burkina Faso, West Africa
Source: Data Brief. 2016 Nov 9;9:1013–9. doi: 10.1016/j.dib.2016.11.001 (PMC5122697; doi:10.1016/j.dib.2016.11.001)
Supplement: Supplementary file 3 — Supplementary material [file mmc3.docx]

| **Project on Climate and Environmental Induced Migration** **in West Africa, Burkina Faso: Climate Risk Perception Questionnaire**  **WASCAL Competence Center, Ouagadougou** |
| --- |
| **1st May, 2014** |

| **Introductory Statement:[VERY IMPORTANT]**  “Scientific evidence have confirmed that the climate will likely change in the future with implications for agriculture, livelihood and food security. The information provided by you in this interview about your perception of climate related risks in your community in the last 5 years will contribute to better understanding of how such risks can be mitigated in the nearest future _________________**[*COMMUNITY:* insert name of country].** We planned to do a follow-up study on this, which will help in the design of better coping mechanisms to risks mitigation in your community. We are therefore requesting that you provide truthful answers to the questions raised. Your responses to these questions will be anonymous.  This study is being conducted by: ____________________________________________________________________  ***(Name of Enumerator)***  Thank you for your kind co-operation.” |
| --- |
|  |
|  |

TS: Time Interview Began:_______ _______

1.0.1 Are you willing or not to participate in the survey? (**KEY for 1.0.1:** 1: Yes; 0: No) ***If No go to 1.0.2 otherwise go to 1.0.3***

1.0.2 Can you explain to us why you are unwilling to participate in the study? (Take down the reasons)

**………………………………………………………………………………………………………………………………………………………………………………………………………………………………………………………………………………………………………………………………………………………………………………………………………………………………………………………………………………………………………………………………………………………………………………………………………………………………………………………………………………………………………………………………………………………………**

1.0.3 Will you be available for the next phase of the interview? (**KEY for 1.0.3:** 1: Yes; 0: No)

1.0.4 Please state the relationship of the respondent to the head of the household:

**KEY for 1.0.4:** 1. Head of household; 2. Husband; 3. Wife; 4: Child; 5. Grandchild; 6. Parents; 7. Siblings; 8. Other family members (includes household helpers). 9: Manager/other proxy for owner

**Section 1: Household Roster--Members of Households and Education**

1.1 Household size (of owner of the farm): (Nos living under same roof)

1.2 Household Characteristics.

|  | 1.2.1 | 1.2.2 | 1.2.3 | 1.2.4 | 1.2.5 | 1.2.6 |
| --- | --- | --- | --- | --- | --- | --- |
|  | Gender | Age  (Years) | Marital status | Education  (in Number of Years) | Work on Farm Activities? | Work on Non-Farm Activities |
| 1 |  |  |  |  |  |  |
| 2 |  |  |  |  |  |  |
|  | **KEY for 1.2.1:**  1: Male  0: Female |  | **KEY for 1.2.3**  1: Married or living together under local custom  2: Never married  3: Previously married (currently divorced, separated, widowed).  4: Not applicable (child < 16 years) |  | KEY for 1.2.5  1: yes  0: no | **KEY for 1.2.6**  1: yes  0: no |

***Please use additional space on back of page if necessary.***

- 1. Which tribe does the household head belong to?

1.4 What religion does the head of the household practice? **KEY for 1.4:** 1. Nonreligious; 2: Christianity; 3: Islam; 4; African Traditional Religion;

5: Other (pls. specify___________)

1.5 Does the household have electricity? (**KEY for 1.5:** 1: yes; 0: no)

We have to have question about the quantity of cereals to feed the household family

1.5a: what quantity of cereals your family needs per year for consumption?

1.5b do you arrive to feed very well your family every year?

**Section 2: Employment All questions pertain to the last 12 months**

| 2.1 | 2.2 | 2.3 | 2.4 | 2.5 | 2.6 | 2.7 |
| --- | --- | --- | --- | --- | --- | --- |
| What is the Primary Occupation of the head of the household | What is the Secondary Occupation of the head of the household | Number of **Days** (per week) spent on Primary Occupation | Number of **weeks** over the last 12 months spent on Primary Occupation | Number of **Days**  (per week) spent on Secondary Occupation | Number of **weeks** over the last 12 months spent on Secondary Occupation | Number of work **days** lost due to illness over the last 12 months? |
|  |  |  |  |  |  |  |
| **KEY for 2.1 and 2.2:**  1. Farmer 2. Agriculture (farm) laborer  3. Artisan 4. Office worker  5. Civil Servant 6. Teacher  7. Health worker 8. Trader  9. Student 10. Unemployed  11. Not in labor force 12. Other non-agriculture worker 13. Fisherman | | *(By definition,*  *one day of work=*  *6-8 hours of work.)* |  | *(By definition,*  *one day of work=*  *6-8 hours of work.)* |  |  |

| **Section 3: Perception of Climate Risk or Hazard** |
| --- |

3.0 How long have you been a farmer (or farm manager)? ______ __(In number of years)

3.1 How long have you been residing in this Community? ______ __(In number of years)

3.2 Have you heard about ‘Climate Hazards’______ __(**KEY for 3.2:** 1: yes; 0: no)

[**Note**: ***If answer is ‘No’ explain the meaning of climate hazard to the respondent and if ‘Yes’, go to 3.2a***]

**‘Climate Hazard’:** An event resulting from changes in weather effects (e.g., temperature and rainfall changes etc) that can lead to negative consequences on all human activities. This may include drought, flooding, strong winds, dust storms, dry spells etc.

3.2a Please can you explain to us what you understand by ‘Climate Hazards’:

**……………………………………………………………………………………………………………………………………………………………………………………………………………………………………………………………………………………………………………………………………………………………………………………………………………………………………………………………………………………………………………………………………………………………………………………………………………………………………………………………………………………………………………………………………………………………………………………………………………………………………………………………………………………………………………………………………………………**

3.3 Please, can you tell us the most common climate-related hazards that affect your community? [**Note**: Take down the list]

**………………………………………………………………………………………………………………………………………………………………………………………………………………………………………………………………………………………………………………………………………………………………………………………………………………………………………………………………………………………………………………………………………………………………………**

3.4 Please, can you also tell us how often these climate-related hazards affect this community?

| Hazards | Frequency in the Last 3 Months | Frequency in the Last 6 Months | Frequency in the Last 1 Years | Frequency in the Last 5 Years | Frequency in the Last 10 Years | Frequency in the Last 30 Years |
| --- | --- | --- | --- | --- | --- | --- |
|  |  |  |  |  |  |  |
|  |  |  |  |  |  |  |
|  |  |  |  |  |  |  |
|  |  |  |  |  |  |  |
|  |  |  |  |  |  |  |
|  |  |  |  |  |  |  |

3.4a [Observation: Please ask if the respondents can recollect any particular climate related hazards in the community]

3.4b How do you compare climate risks to other risks (1 = the worst, 2 = worse, 3 = very moderate, 4 = moderate, and 5 = do not know)

3.5 Please, can you to rank these hazards from the most important to the least important as they affect your community?

| **List of Hazards** | **Ranking last 1 Year** | **Ranking last 5 Year** | **Ranking last 10 Year** | **Ranking last 10 Year** |
| --- | --- | --- | --- | --- |
|  |  |  |  |  |
|  |  |  |  |  |
|  |  |  |  |  |
|  |  |  |  |  |
|  |  |  |  |  |
|  |  |  |  |  |

[**Note Enumerator]**

**3.5a**  Please, starting with the highest ranking hazard and covering each of the other hazards, ask the respondent to indicate why the ranking was given:

**………………………………………………………………………………………………………………………………………………………………………………………………………………………………………………………………………………………………………………………………………………………………………………………………………………………………………………………………………………………………………………………………………………………………………………………………………………………………………………………………………………………………………………………………………………………………………………………………………………………………………………………………………………………………………………………………………………………**

**………………………………………………………………………………………………………………………………………………………………………………………………………………………………………………………………………………………………………………………………………………………………………………………………………………………………………………………………………………………………………………………………………………………………………………………………………………………………………………………………………………………………………………………………………………………………………………………………………………………………………………………………………………………………………………………………………………………**

**………………………………………………………………………………………………………………………………………………………………………………………………………………………………………………………………………………………………………………………………………………………………………………………………………………………………………………………………………………………………………………………………………………………………………………………………………………………………………………………………………………………………………………………………………………………………**

3.6 Have you experience any direct or indirect outcomes of these hazards on your farm? .......... (**KEY for 3.6:** 1: yes; 0: no)

[**Note**:] If answer is yes, record for each hazard as provided by the respondent.

| **Hazards** | **Direct or indirect outcomes of hazard on farmland** |
| --- | --- |
|  |  |
|  |  |
|  |  |
|  |  |
|  |  |
|  |  |

3.7 Can you remember when you experienced the last hazard on your farm? (**KEY for 3.7:** 1: Yes; 0: No)

[**Note**:] If answer is **Yes**, ask the respondent to explain the nature of hazard.

**………………………………………………………………………………………………………………………………………………………………………………………………………………………………………………………………………………………………………………………………………………………………………………………………………………………………………………………………………………………………………………………………………………………………………………………………………………………………………………………………………………………………………………………………………………………………………………………………………………………………………………………………………………………………………………………………………………………**

3.8 What was the total damage in terms of crops? (In kilograms or Per Farm Hectare)

[**Note**: This only refers to most recent episode of hazard of farm}

| *List of Crops* | Damage in Kilogram | Damage Per Hectare |
| --- | --- | --- |
| Crop 1: |  |  |
| Crop 2: |  |  |
| Crop 3: |  |  |
| Crop 4: |  |  |
| Crop 5: |  |  |

3.8a During which period of the farming season did the climate hazard occurred?

| List of Crops | **Exact Farming Season that Damage Occurred** |
| --- | --- |
| Crop 1: |  |
| Crop 2: |  |
| Crop 3: |  |
| Crop 4: |  |
| Crop 5: |  |

3.9 If you were to be compensated for the total crop damage, how much would you ask for? (Total Cost of Damage)

3.9a Can you explain your reasons for asking this amount?

**………………………………………………………………………………………………………………………………………………………………………………………………………………………………………………………………………………………………………………………………………………………………………………………………………………………………………………………………………………………………………………………………………………………………………………………………………………………………………………………………………………………………………………………………………………………………………………………………………………………………………………………………………………………………………………………………………………………**

| **Section 4: Effects of Climate Change** |
| --- |

4.0 How do you perceive nowadays season onset and offset compare to that of 10, 20 and 30 years ago?

………………………………………………………………………………………………………………………………………………………………………………………………………………………………………………………………………………………………………………………………………………………………………………………………………………………………………………………………………………………………………………………………………………………………………………………………

4.1 How do you perceive nowadays dry spell frequency compare to that of 10, 20 and 30 years ago?

…………………………………………………………………………………………………………………………………………………………………………………………………………………………………………………………………………………………………………………………………………………………………………………………………………………………………………………………………………………………………………………………………………………………………………………………………………………………………………………………………………………………………………………………………………………………………………………………

4.2 Describe the dry spell damage to crops in relation to growing stage (sowing, flowering and harvesting)

………………………………………………………………………………………………………………………………………………………………………………………………………………………………………………………………………………………………………………………………………………………………………………………………………………………………………………………………………………………………………………………………………………………………………………………………………………………………………………………………………………………………………………………………………………………………………………………………

4.3 List the principal factors that cause “bad” crop yields

……………………………………………………………………………………………………………………………………………………………………………………………………………………………………………………………………………………………………………………………………………………………………………………………..

4.4 During the last episode of the climate-related hazard that occurred in your community, did your household felt secured or not? ……………… (**KEY for 3.2:** 1: yes; 0: no) [**Note**:] If answer is yes, go to **4.1** and if no, ask respondent to explain why the insecurity].

4.5 Explain the cause of the insecurity?

**…………………………………………………………………………………………………………………………………………………………………………………......**

**…………………………………………………………………………………………………………………………………………………………………………………......**

4.6 What methods do you normally employ to reduce the occurrence of these hazards on your farm area? [**Note**: List action taken for each hazard]

| **Hazard** | **Management Actions taken by Households** |
| --- | --- |
|  |  |
|  |  |
|  |  |
|  |  |
|  |  |
|  |  |
|  |  |
|  |  |

4.6a For each of the management methods can you tell us how much was the total cost to your household?

| **Hazard** | **Management Actions** | **Cost to Household** |
| --- | --- | --- |
|  |  |  |
|  |  |  |
|  |  |  |
|  |  |  |
|  |  |  |
|  |  |  |

| **Instructions: This section to be filled out by interviewer.** | | **Time Interview Ended:______** **_____** | | | | | |
| --- | --- | --- | --- | --- | --- | --- | --- |
|  |  |  |  |  |  |  | |
| Name of interviewer:________________________________ (this information is important to validate survey responses and will be used to cross check in the event that there are unusual observations during the analysis of the data) | | | | | | | |
|  |  |  |  |  |  |  | |
| Date of interview (mm/dd/yr) |  |  |  |  |  | **Location of Farm** | |
|  |  |  |  |  |  |  |  |
| Respondent Households’ identification number  (Unique Household ID- should be assigned prior to interview) |  |  |  |  |  | Country |  |
| (1=interview 1, 2=interview 2,…….n=survey n) for each district |  |  |  |  |  |  |  |
|  |  |  |  |  |  | Province/Region |  |
|  |  |  |  |  |  |  |  |
|  |  |  |  |  |  | District |  |
|  |  |  |  |  |  |  |  |
|  |  |  |  |  |  | District Code  (as provided by YALE) |  |
|  |  |  |  |  |  |  |  |
|  |  |  |  |  |  | Subdivision/Division |  |
|  |  |  |  |  |  |  |  |
|  |  | | | |  | Village |  |
|  |  |  |  |  |  |  |  |

***Optional: Contact Information of Respondent:__________________________________________________________________***

***(address) __________________________________________________________________***
