# Supplementary material for: Survey data on key climate and environmental drivers of farmers’ migration in Burkina Faso, West Africa
Source: Data Brief. 2016 Nov 9;9:1013–9. doi: 10.1016/j.dib.2016.11.001 (PMC5122697; doi:10.1016/j.dib.2016.11.001)
Supplement: Supplementary file 4 — Supplementary material [file mmc4.docx]

| **Climate and Environment Induced Migration Interview Guide and Focus Group Discussion Topics in West Africa, Burkina Faso.**  **WASCAL Competence Center, Ouagadougou** |
| --- |

**INTERVIEW GUIDE N°1 (migrant farmers)**

**IDENTIFICATION**

| Last name: |  | Marital status: |
| --- | --- | --- |
| First name: |  | Religion: |
| Gender: |  | Seniority: |
| Organization: |  | Interview duration: |
| Habitual residence: |  | Interview date: |
| Work place: |  | Interviewer name: |
| Level of Education:  Age: |  | Interview number:  Tel: |

**Perception of the climate change and the agricultural practice**

1. According to you, who is a farmer? (probe his definition of practice); and what is his place and his function in society?
2. How can we recognize a 'good' farmer?
3. How do you see climate change?
4. Area (potential) of specialization in agriculture (probe: Subsistence crops or Cash crops)
5. Origin of knowledge (probe: mode of agricultural knowledge acquisition) Location of the field

**Agricultural practices and migration**

1. What are your areas of activity? (Probe: only agriculture or other additional activities)?
2. Do you practice agriculture as at the time of your parents (or grandparents?) or did you change your farming way?
3. What has changed in your practices? (probe: what he has abandoned or improved) Why? (examples)
4. Why have you chosen to practice agriculture here? (probe his/her sense of migration in agriculture)
5. Have you already migrated to another location before? Why? (examples)
6. What do you think about the quality and the use of the land here?
7. Is it important to preserve the land? (probe: what is the meaning and usefulness of the land in their activity)
8. If you are a migrant, what can you say about the climate conditions of your host zone compared to your starting area? (probe his/her findings on climate)

1. Are your yields related to climatic hazards or other requirements? (probe his/her social and economic relationships with other farmers)?
2. Can the fact to abandon his/her customs affect one’s activities? (probe: threats of progressive loss of the material and agricultural productivity)
3. Generally, what differentiates an indigenous farmer to a migrant? (probe: the mode of acquisition of the land, the conditions of production and agricultural products distribution).
4. Did you come in group or individually? What were your motivations at the beginning? Are they always the same? If not, why?

1. How do you see the future of agriculture?
2. How do you see your own future in agricultural practice?

What is your last word?

Thank you for your collaboration

**INTERVIEW GUIDE N°2 (indigenous farmers)**

| Last name: |  | Marital status: |  |
| --- | --- | --- | --- |
| First name: |  | Religion: |  |
| Gender: |  | Seniority: |  |
| Organization: |  | Interview duration: |  |
| Habitual residence: |  | Interview date: |  |
| Level of Education: |  | Interviewer name: |  |
| Age: |  | Interview number:  Tel: |  |
|  |  |  |  |

**Farming perception and climate change**

1. According to you, who is a farmer? (probe his/her definition of the practice): and what is his place and his function in the society?
2. How do you see the climate change?
3. How do you recognize a ‘good’ farmer under climate change?
4. Area (potential) of specialization (probe : subsistence crops or cash crops)

Knowledge origin (probe: mode of acquisition of his/her agricultural knowledge)

Field location

**Agricultural practices and migration**

1. What are your areas of activity? (probe: only agriculture or other additional activities)
2. Do you practice agriculture as at the time of your parents (or grandparents?) or did you change your farming way?
3. What has changed in your practices? (probe: what he/she has abandoned or improved) Why? (examples)
4. What have you kept in your practices? Why? (examples)
5. Have you already migrated as a farmer?

If not, what aspects have kept you in the same place so far? (probe: climate conditions, agricultural practices)

1. What meaning do you give to land and agricultural practice?
2. Is it important (or not) to preserve land and to keep the same agricultural practices? How?
3. How do you perceive farmers who migrate? (probe : his cohabitation and his collaboration with migrants)
4. What difference are there between them (migrants) and you (indigenous)?
5. Are you willing to follow your farming activities under the current climate conditions? Why? (probe : advantages and disadvantages of climate change)
6. How do you see the future of agriculture?
7. How do you see your own future in agriculture practice?

What is your last word?

Thank you for your collaboration

**INTERVIEW GUIDE N°3 (Resource persons)**

**IDENTIFICATION**

| Last name: |  | Interview duration: |  |
| --- | --- | --- | --- |
| First name: |  | Interview date: |  |
| Gender: |  | Interviewer name: |  |
| Structure: |  | Interview number: |  |
| Level of Education: |  | Tel: |  |

Age:

1. There is, since some few years, a strong finding of the climate change: how do you appreciate this phenomenon in the Dano commune?
2. What observation do you do about the evolution of the current practices of agriculture stakeholders? (probe: farmers, technicians and engineers)
3. What were your roles in this process?
4. Apart from the actions already taken, what do you plan for improvement?
5. Are your methods adopted by farmers? If yes, by which categories (migrants or indigenous). If not, why?
6. How do you perceive the migration movement of farmers to Dano?
7. What is the estimated number of migrants per year? At this time?
8. How did this process begin and what does it mean for you?
9. What are its advantages and constraints?
10. What are the objectives and the main orientations of the current climate change policy of Burkina Faso?
11. What are your achievement in the supervision and the monitoring of climate change and farmers?
12. What are your difficulties and constraints?
13. How do you see the future of agriculture under the current climate conditions?

What is your last point?

Thank you for your collaboration

**Focus Group Discussion Topics**

Topic 1. Local indicators of climate change.

Topic 2: Climate risks to agriculture.

Topic 3: Local strategies to adapt to climate change and food scarcity.
