# Supplementary material for: Survey data on key climate and environmental drivers of farmers’ migration in Burkina Faso, West Africa
Source: Data Brief. 2016 Nov 9;9:1013–9. doi: 10.1016/j.dib.2016.11.001 (PMC5122697; doi:10.1016/j.dib.2016.11.001)
Supplement: Supplementary file 5 — Supplementary material [file mmc5.docx]

Dear editorial board of Data in Brief,

Please find enclosed the revised manuscript: “Survey Data on Key Climate and Environmental Drivers of Farmers’ Migration in Burkina Faso, West Africa”, by Safiétou Sanfo, M. William Fonta, Boubacar Ibrahim and John P.A. Lamers to be submitted as a data article to Data in Brief for consideration of publication. All co-authors have seen and agree with the contents of the manuscript. We certify that the submission is original work and is not under review at any other publication.

In this manuscript, we describes two datasets generated from various sources in southwestern Burkina Faso to identify the key environmental drivers that cause farmers to migrate.

We believe that our findings could be of interest to the readers of Data in Brief. Analyses of these data may allow for comparisons between this sample and parallel samples in other similar studies elsewhere, namely in other West African countries.

We hope that the editorial board will agree on the interest of this data article.

Sincerely yours,

Safiétou Sanfo on behalf of the authors.

**Corresponding author**: Safiétou Sanfo, agricultural economist, WASCAL (West Africa Science Service Center on Climate Change and adapted Land Use); 06 BP 9507, Ouagadougou 06, Burkina Faso. [safi.sanfo@wascal.org /](mailto:safi.sanfo@wascal.org/) [safi.sanfo@laposte.net](mailto:safi.sanfo@laposte.net) phone number: office +22650375423 / cell: +22679264850.
